# Photodynamic Therapy in Acne Vulgaris: A Systematic Review

**DOI:** 10.1177/12034754241291031

**Published:** 2024-11-18

**Authors:** Simal Qureshi, Zahra Rehan, Aggie Ao, Ilya Mukovozov

**Affiliations:** 1 Faculty of Medicine, Memorial University of Newfoundland, St. John’s, NL, Canada; 2 Cumming School of Medicine, University of Calgary, Calgary, AB, Canada; 3 Faculty of Health Sciences, University of Western Ontario, London, ON, Canada; 4 Toronto Dermatology Centre, Toronto, ON, Canada

**Keywords:** acne, photodynamic therapy (PDT)

## Abstract

Acne is a multifactorial disorder of the pilosebaceous unit. Photodynamic therapy (PDT) is an energy-based treatment shown to be safe in acne vulgaris, although the mechanism of action of PDT in acne is incompletely understood. This review summarizes the clinical features of and treatment efficacy in acne patients treated with PDT. A systematic review of Medline, Embase, and the Cochrane Database of Systematic Reviews was conducted. Title, abstract, full-text screening, and data extraction were completed using Covidence. Studies reporting the use of PDT in patients with acne were included while clinical features and treatment responses were extracted. Treatment outcomes were scored as complete response, partial response, and no response. After screening a total of 1122 studies, 82 studies met the inclusion criteria, representing 56 prospective studies, 25 randomized controlled trials, and 1 retrospective study. Results representing 4340 patients with acne (mean age 24.4 years; 52% females) treated with PDT are summarized. Overall, 2154 (50%) participants underwent aminolevulinic acid PDT, 452 (10%) participants underwent methyl aminolevulinate PDT, 28 (<1%) participants underwent daylight PDT, and 1706 (39%) underwent other modalities of PDT. The average follow-up period after study completion was 3 months, ranging from 2 weeks to 13 months. A partial response was observed in treated patients with outcome measures including clinical response, lesion count, pain, recurrence, and patient satisfaction. This systematic review provides preliminary data summarizing the clinical features and treatment efficacy in patients with acne treated with PDT. Our results suggest a partial clinical response when using PDT to manage acne. Future studies should focus on standardizing study protocols and drawing direct comparisons between PDT and other modalities for acne treatment.

## Introduction

Acne is a multifactorial disorder of the pilosebaceous unit and a chronic inflammatory condition affecting individuals of all ages, most commonly in individuals 12 to 25 years of age.^1^ Along with the substantial personal burden experienced by patients, acne vulgaris accounts for considerable costs to the health care system. Among dermatologists and family physicians, acne is the most common diagnosis.^2^ The pathophysiology includes increased sebum production, altered keratinization, bacterial proliferation, and inflammation in the pilosebaceous unit.^3^ Abnormal keratinization leads to the development of enlarged progressive hyperkeratotic plugs that form closed or open comedones. A higher level of circulating androgens leads to increased sebum production and contributes to the development of comedones.^2^ Additionally, *Cutibacterium acnes* (*C. acnes*) is a normal component of the skin microbiota, which flourishes using sebum as a nutrient source.^4^ With increased sebum production, *C. acnes* thrives and plays a key role in inflammatory processes, such as papules, pustules, nodules, or cysts.^5^ There are inflammatory reactions in the cutaneous environment that foster acne lesion progression as a result of multifactorial processes.

In photodynamic therapy (PDT), a reaction occurs from the interaction between a chemical photosensitizer and a specific wavelength of light in the presence of oxygen. Light activation produces therapeutic benefits as the accumulation of porphyrins causes the formation of potent oxidizers.^6^ Examples of photosensitizers include methyl aminolevulinate (MAL), aminolevulinic acid (ALA), indocyanine green, and methylene blue. These photosensitizers are often combined with light sources such as red light, blue light, and intense pulsed light. Many factors may impact the efficacy of PDT including skin preparation, concentration of the photosensitizer, time between application and light exposure, tissue oxygen availability, and the wavelength of the light source.^6,7^

Treatments to manage acne include topical retinoids, topical and oral antibiotics, and isotretinoin.^8^ However, these treatments can cause skin irritation and often require daily use leading to poor patient adherence.^8^ As an alternative or adjunct to traditional treatments for acne vulgaris, PDT utilizes a specific wavelength of light to target sebaceous glands leading to improvement in acne.^9^ Prior studies have shown that PDT may decrease the size of sebaceous glands and cause immunological changes in the pilosebaceous unit leading to improvement in acne burden.^6,10,11^

Currently, there is a lack of systematic reviews on clinical features and efficacy in PDT for acne patients. Therefore, this study aimed to summarize the type of PDT employed, clinical features of treated patients, and outcomes including efficacy, complications, and follow-up.

## Methods

A systematic review of the literature was conducted adhering to PRISMA reporting guidelines.^12^ The study protocol was registered with the PROSPERO database (CRD42023461631).

### Study Eligibility Criteria

Study eligibility was identified using the Population, Intervention, Comparator, Outcomes, and Study Design (PICOS) criteria, a framework used to encapsulate the purpose and key components of a review (cite). Studies were selected if articles met the following PICOS criteria:

- Population: All individuals diagnosed with acne regardless of age and sex; including any disease, severity, treatment received, or comorbid conditions.
- Intervention: Photodynamic therapy; including conventional and daylight PDT and any photosensitizer.
- Comparator: Untreated individuals with acne; studies on outcomes assessed in treated individuals with acne without a comparator were included.
- Outcomes: Pain, lesion count, Visual Analogue Scale (VAS), Dermatology Life Quality Index, recurrence, healing time, physician global assessment, patient satisfaction, and clinical response.
- Study design: Observational studies including prospective and retrospective cohort, cross-sectional, case-control studies, and case series.

### Literature Search and Screening

The search was conducted using the OVID interface with a focus on publications between 1946 utilizing Medline and from 1974 utilizing Embase to July 5, 2020. Literature within the study was searched in the Cochrane Database of Systematic Reviews and the PubMed database, in addition to hand-searched references. Title, abstract, and full-text screening were performed independently by 2 reviewers (S.Q. and Z.R.) using Covidence (www.covidence.org). Eligible articles were selected within the full-text screening process if met the predetermined PICOS criteria. Conflicts between reviewers during the selection process were resolved through discussion with the senior author until a consensus was reached. There are no language limitations during the search.

### Data Extraction

Data extraction was completed by 2 reviewers utilizing a standardized extraction form (S.Q. and Z.R.). Extracted variables included the following: study characteristics (publication year, study design, and sample size), patient characteristics (age and sex of participants), the severity of acne, interventions used, the patient’s response to treatment, and outcome measures (complete, partial, or no clinical response, lesion count, patient satisfaction, VAS, pain, and recurrence).

### Risk of Bias (Quality) Assessment

The Joanna Briggs Institute (JBI) critical appraisal tool for case series was used to assess the risk of bias of included studies by 2 reviewers (S.Q. and Z.R.).^13^ Bias assessments were completed independently by each reviewer prior to the discussion of conflicting studies. The JBI criteria included 10 questions to assess the following: (1) whether there was a clear inclusion criterion, (2) whether the condition was measured in a standardized and reliable process for all patients, (3) whether valid methods were used to identify the conditions for all participants, (4) whether there was a consecutive inclusion of participants, (5) whether there was a complete inclusion of participants, (6) whether there was a clear reporting of patient demographics, (7) whether there was a clear reporting of patient clinical information, (8) whether the outcomes or follow-up results were reported, (9) whether clear reporting of presenting site(s) or clinic(s) information were included, and (10) whether statistical analysis was appropriate.^13^ Each of the questions was answered with “yes,” “no,” or “unclear” by the 2 reviewers. Nine of the questions were included; however, the tenth question was deemed “not applicable” and was excluded. The included studies were assessed based on their risk of bias and were grouped as follows: (1) low risk of bias (studies that met 7-10 of the quality criteria), (2) moderate risk of bias (studies that met 4-6 of the quality criteria), and (3) high risk of bias (studies that met less than or equal to 3 quality criteria).

## Results

### Study Characteristics

After the title and abstract screening of 1122 articles, 82 articles were included in the systematic review as shown in Supplemental Figure S1. Studies were published between 2000 and 2021, with 56 prospective studies, 25 randomized controlled trials, and 1 retrospective study. The most common topical photosensitizers used across the studies were 5-aminolevulinic acid, methyl aminolevulinate, and indocyanine green. The 3 topical photosensitizers demonstrate comparable results. The light sources showed similar efficacy with the common utilization of red light, blue light, and intense pulsed light. Within all studies, the most prevalent outcome measured was lesion count with inflammatory lesions responding well to treatment shown by a partial response counted. Patients with Fitzpatrick skin types I to VI were included across multiple studies. Adverse events associated with PDT for acne such as burning sensations, pain, itching, blisters, pruritus, hyperpigmentation, and erythema were present.

### Patient Characteristics

The total analysis included 4340 patients, with 56% (n = 2253) females and a mean age of 24.4 years. Of the studies included, 2154 (50%) participants underwent ALA-PDT, 452 (10%) participants underwent MAL-PDT, 28 (<1%) participants underwent daylight PDT, and 1706 (39%) underwent other modalities of PDT. Mild acne was present in 0.7% of the study participants, moderate acne presented in 10.2%, and severe acne presented in 25.6%. For participants with a range of acne severity, 37.4% had mild-moderate acne, 20.4% had moderate-severe acne, and 5.7% had mild-severe acne.

### Treatment Response

A partial response was observed in treated patients, with no patients reporting a complete or lack of response to the treatment. Included studies used a variety of outcome measures such as lesion count, patient satisfaction, photography, global assessment scale, or VAS to measure the efficacy of the treatment. In summary, 79 studies measured outcomes using lesion count, 17 studies used photography, 7 studies measured patient satisfaction levels, 5 studies used the global assessment scale, 4 studies used the VAS, 4 studies used the investigator global assessment, and 2 studies measured sebum secretion levels. The average follow-up duration after study completion was 3 months, with follow-up periods ranging from 2 weeks to 13 months.

### Quality Appraisal

Based on the JBI Critical appraisal, the overall mean quality appraisal score for the studies included in this review was 5.8 (moderate risk of bias), ranging from a high risk of bias of 2 to a low risk of bias of 9. More specifically, the quality appraisal yielded moderate risk for 56.1% of studies, followed by low risk for 35.4% and high risk for 8.5%.

## Discussion

Acne vulgaris is a common chronic skin condition, which can lead to scarring and hyperpigmentation, resulting in negatively impacting a patient’s mental health. In the pathogenesis of acne, an increase in sebum production that can obstruct a hair follicle containing keratin, sebum, and normal flora is known as comedones.^14^ The accumulation and inflammation of comedones can lead to the formation of acne papules and pustules.^14^ *C. acnes* contributes to inflammatory factors of tumour necrosis factor-α (TNF-α), interleukin-6 (IL-6), IL-8, and IL-12. Therefore, the release of the inflammatory mediators leads to the development of papules and pustules.^7,14^

The standard acne treatments are benzoyl peroxide, topical retinoids, oral isotretinoin, and oral and topical antibiotics. Medication failure, intolerable side effects, and a lack of adherence are present in standard treatment for acne.^15^ Prior research had suggested PDT to be a promising treatment for acne management.^8^ It has been verified that direct or high-dose PDT causes injury to the sebaceous gland, thus inhibiting serum secretion. *C. acnes* are destroyed by ALA-PDT sterilization of sebaceous follicles, which effectively reduces follicular obstruction through altering keratinocyte shedding and hyperkeratosis.^7,16,17^ The pilosebaceous units of acne include pathogenic species such as *C. acnes*, *S. epidermidis*, and *P. fluorescens*.^15^ ALA-PDT is shown to be more effective in inactivating gram-positive bacteria such as *C. acnes* and *S. epidermidis*.^18^ This mechanism of action consists of inhibiting microbiota function, therefore increasing *P. fluorescens* and cultivating a healthy microbial community through microbiome diversity in pilosebaceous units.^18,19^

An abundance of data in the literature supports PDT as an efficacious treatment for acne.^6,8-11,20,21^ For example, a randomized controlled trial by Zhang et al^20^ reported 5.2%, 29.3%, 78.3%, and 85.7% efficacy following the patient’s completion of the first, second, third, and fourth cycles of ALA-PDT, respectively. Bissonnette et al,^21^ a single-centre, randomized, and single-blind study, indicated a decrease in lesion inflammation post-treatment with MAL-PDT with patients tolerating treatment, and no serious adverse events were documented. Additionally, Boen et al^7^ found statistically significant response rates with ALA-PDT and MAL-PDT and a decrease in inflammatory lesion counts with treatment within a few high-quality randomized placebo-controlled trials. The findings of this study are similar to previously published literature, with a vast majority of studies reporting a decrease in lesion inflammation contributing to higher efficacy and response rates. Despite the evaluation of PDT as the sole intervention of our systematic review, PDT can be an adjunct therapy with topical retinoids or laser and energy-based device treatments.^8^

This systematic review concludes that PDT can be effective in the treatment of acne, with evidence supporting partial improvements across various treatment parameters.^22^ All studies demonstrated a clinical improvement that was signified by a partial response; however, there were no studies that reported a complete response. There was inconsistency with using identical outcomes across studies, but multiple studies used various outcomes to measure a partial improvement. Furthermore, the administration of PDT with varying numbers of treatment sessions, incubation times, and total energy doses of the light source was inconsistent. The trend toward shorter incubation times and low-strengthening photosensitizers to decrease adverse side effects was present. Moving forward, repeating trials with a fixed outcome and consistent parameters will strengthen the accuracy of estimating the efficacy of PDT.

The latest acne management guidelines make no recommendations regarding the use of PDT as a treatment modality.^23^ However, clinical improvements have been demonstrated with the use of PDT including the decreased presence of lesion inflammation, visual improvement indicated through photography, and the increased satisfaction of patients.^20^ With that said, there is a minimal reduction in the number of non-inflammatory lesions.^20^ Patients who have experienced pain and side effects such as erythema, pustular eruption, epithelial exfoliation, and hyperpigmentation, during the application process of the treatment were reported.^24^ The exploration of the adaptation of PDT as a modality for acne treatment to decrease pain and minimize side effects is required to deliver effective and long-lasting clinical outcomes.

### Limitations

There are several limitations to this systematic review. Firstly, all studies yielded a partial response as opposed to none or complete response in the involvement of assessing the efficacy of PDT. There is likely a considerable heterogeneity within this group when comparing mild partial response to strong partial response. Secondly, several of the included studies primarily focused on patient satisfaction as their outcome measure. Since this relies on an individual’s subjective judgment, the efficacy of PDT on acne is affected. Additionally, there was a challenge in forming comparisons and pooling the data from different studies due to the considerable heterogeneity in the acne severity of participants in the included studies. The difference in outcome measures for acne severity in post-treatment was challenging to form conclusive comparisons. Further, the majority of included studies were open-label studies evaluating PDT in acne patients, so direct comparisons with other therapies are lacking. Lastly, the protocols for PDT such as photosensitizers and light sources varied across studies. Due to the differing variables and a lack of definitive criteria or guidelines, it results in a gap in the literature to develop more streamlined studies that focus on identifying the most effective variables and parameters for the safe and efficacious use of PDT for acne.

Future studies assessing the efficacy of PDT for acne can be strengthened by refining study methodologies and incorporating direct comparators, such as topical products.

## Supplemental Material

sj-tif-1-cms-10.1177_12034754241291031 – Supplemental material for Photodynamic Therapy in Acne Vulgaris: A Systematic Review Supplemental material, sj-tif-1-cms-10.1177_12034754241291031 for Photodynamic Therapy in Acne Vulgaris: A Systematic Review by Simal Qureshi, Zahra Rehan, Aggie Ao and Ilya Mukovozov in Journal of Cutaneous Medicine and Surgery
